# Patterns of passage into protected areas: Drivers and outcomes of Fulani immigration, settlement and integration into the Kachia Grazing Reserve, northwest Nigeria

**DOI:** 10.1186/s13570-017-0105-1

**Published:** 2018-01-11

**Authors:** Marie J. Ducrotoy, Ayodele O. Majekodunmi, Alexandra P. M. Shaw, Husein Bagulo, Wilson J. Bertu, Amahyel M. Gusi, Reuben Ocholi, Susan C. Welburn

**Affiliations:** 1 Division of Infection and Pathway Medicine, School of Biomedical Sciences, College of Medicine and Veterinary Medicine, The University of Edinburgh, Chancellor’s Building, 49 Little France Crescent, Edinburgh, EH16 4SB UK; 2 Livestock and Poultry Research Centre, College of Basic and Applied Sciences, University of Ghana, P. O. Box LG 38, Legon, Ghana; 3 Avia-GIS, Risschotlei 33, B-2980 Zoersel, Belgium; 4 Brucellosis Research Unit, National Veterinary Research Institute, P.M.B. 01, Vom, Plateau State 930001 Nigeria; 5 Present Address: Ceva Santé Animale, 10 Avenue de la Ballastiere, 33500 Libourne, France

**Keywords:** Fulani, Nigeria, Pastoralism, Immigration, Conflict

## Abstract

Increasing land use and associated competition for natural resources in the wake of high human and livestock population pressures have been major challenges confronting pastoralists of West Africa. This is especially true in Nigeria where Fulani make up 4% of the national population and prevailing national insecurity issues are impacting on pastoral livelihoods, including violent conflicts over land and ethnic, religious and political disparities.

This study examined the dynamics of immigration within the Kachia Grazing Reserve (KGR), an exclusively Fulani pastoralist community in Kaduna State, northwest Nigeria, prompted by concerns from both the farming communities and the authorities about mounting pressure on existing limited resources, particularly in regard to availability of cattle grazing resources.

Drawing from a household census conducted in 2011 and employing a range of qualitative methods (focus group discussions and key informant interviews), this study explored the drivers and consequences of immigration and subsequent integration within the KGR community.

The study revealed two types of immigration: a steady trickle of pastoralists migrating to the reserve to settle and acquire land, secure from the stresses of competition from cultivators, and the sudden influx of internally displaced persons fleeing violent clashes in their areas of origin.

Population pressure within the reserve has risen steadily over the past three decades, such that it is severely overgrazed (as evidenced by reports from the KGR community that the animals run short of pasture even during the wet season due to desertification and the spread of non-edible weeds). The newer immigrants, fleeing conflict, tended to arrive in the reserve with significantly larger herds than those kept by established residents. Pastoralists in the reserve have been forced back into the practice of seasonal transhumance in both wet and dry seasons to support their herds, with all the attendant risks of theft, clashes with cultivators and increased disease transmission.

## Introduction

Fulani pastoralists are widely distributed across West Africa and represent the largest migratory ethnic group in the world. The origin of the Fulani is obscure (Ibrahim 1966). The genetic and linguistic evidence points to a West African origin in Senegambia (Scheinfeld et al. 2010, Cruciani et al. 2010, Winters 2010; Cerny et al. 2006, Tishkoff et al. 2009). It is suggested that they moved eastwards in search of pasture for their cattle, passing through Messina and the Hausa states towards Chad, eventually reaching Sudan (Blench 1994). Stenning (1957) reports that their spread through gradual migratory drift over many centuries was interspersed with periods of intense migration associated with Islamic holy wars (*jihad)*.

Nigeria has a long history of pastoralism. The Fulani are thought to have reached Nigeria (Hausaland) in the thirteenth century, by which time they had embraced Islam (Ibrahim 1966). By the fifteenth century, some Fulani had largely abandoned herding and settled to become scholars and counsellors in the courts of the Hausa rulers (Waters-Bayer and Bayer 1994). The rest maintained seasonal migration with their herds, relying on the ‘settled’ elite Fulani to ensure right of passage and access to pasture. In the nineteenth century, Usman Dan Fodio led a jihad against the Hausa rulers, securing his Fulani leadership and supremacy in much of northern Nigeria. At this point, the tribe divided into the Fulani elite who intermarried with the Hausas and the ‘cattle Fulani’ or *Bororo* who continued with their pastoral life (Ibrahim 1966).

Fulani account for 4% of the national population of Nigeria (World Atlas 2017). Fulani pastoralists still dominate cattle production in Nigeria, mostly as transhumant agro-pastoralists practicing seasonal movements of cattle from a permanent base (Azuwike and Enwerem 2010; Iyayi et al. 2003; Blench 2001). The Fulani in Nigeria include the following: settled Fulani (many of whom are descendants of the aristocratic elite of what was previously known as Hausaland), semi-sedentary Fulani (often agro-pastoralists) and a minority group of nomadic pastoral Fulani subsisting entirely off their herds.

Large populations of pastoralists are gradually emigrating from their prior grazing grounds in the north of Nigeria. Changes in land use, expansion of agricultural activities, population growth, climate change and desertification have progressively pushed pastoralists south as the savannahs become less favourable environments for both farmers and pastoralists (Blench 1996; Roma 2008; Mwiturubani and van Wyk 2010, Young and Goldman 2015). Pastoralists are also pulled south by adaptive features such as the establishment of social networks, herding contracts, dairy development and cattle cross-breeding programmes (Bassett and Turner 2007) - for example, the public-private Dairy Development Programme of Oyo State, https://www.frieslandcampina.com.ng/sustainability/dairy-development/).

Pastoralists often relocate voluntarily, attracted by abundant natural resources, greater security, reduced population pressure, and better opportunities of education for their children and trade. Nigeria accounts for 50% of the beef consumed in West Africa with consumption estimated at 433 thousand tonnes in 2015 (https://data.oecd.org/agroutput/meat-consumption.htm). Demand is mostly concentrated in affluent and populous southern cities such as Lagos, Ibadan and Port Harcourt where most large cattle markets and abattoirs are situated (Bénard et al. 2010). The cattle markets of southern Nigeria are no longer simply places where cattle exchange hands. The lands surrounding these trading hubs have now turned into major grazing bases and havens protecting pastoralists from irate crop farmers in the north (Azuwike and Enwerem 2010).

There has been a steady incursion of pastoralists into the humid and sub-humid zones of Nigeria over at least the past five decades (Bourn et al. 2001). Clashes between herders and farmers are often as a result of the destruction of crops in cultivated farmlands by cattle, obstruction of traditional migration routes and livestock theft. There is mutual distrust, and each group blames the authorities for favouring the other side. For example, the plan presented in January 2016 to the Nigerian Governors Forum by the government to map grazing areas in all states as a temporary solution for cattle owners was vehemently opposed by most central and southern states who viewed it as favouring Fulani herders. To mollify this opposition, a bill was sent to the National Assembly by the Agriculture Minister in March 2016 to prohibit cattle from roaming in cities and villages, a decision very badly received by the Fulani community (International Crisis Group 2017).

Both sides, however, have their share of the blame in instigating violence, murders and damage to property or resources. For example, Bukar (2016) investigating management between farmers and pastoralists in Borno State found that farmers reported a lot of negative stories of migrant pastoralists deliberately allowing animals into cultivated fields, careless cutting of trees and violent behaviour when confronted. Baidoo (2014) in his case study of Fulani herders and farmers in the Ashanti region (Ghana) reported that spraying of farmland with pesticides, cultivating crops close to kraals and river banks where cattle go to drink water, encroachment upon grazing routes and shooting of cattle that stray onto farmland was seen by Fulani herdsmen as a deliberate attempt to kill their cattle and reduce their access to grazing pasture. Raping of women by Fulani herdsmen was also a notable cause of the strained relationship (Baidoo 2014).

Pastoralists, by virtue of their mobility, can flee insecurity in more volatile areas to go to locations where political tension and violence are less rife. This is in contrast to sedentary agriculturalists, who have more to lose (i.e. land) by relocating.

Transhumant and nomadic pastoral Fulani would argue that their interests with regard to land rights and rights of passage during transhumance are largely neglected by the state and this has led to frustration and conflict (Blench et al. 2006).

This study explored the drivers and consequences of immigration into the Kachia Grazing Reserve (KGR), Kaduna State, and subsequent integration within the KGR community.

### Farmer-herder relations in Nigeria

A symbiotic relationship has traditionally existed between pastoralists, crop farmers and their environment in Nigeria. The vegetation zones of Nigeria from north to south include (1) Sahel (dry zone - camels and goats), (2) Sudan savannah (lighter woodland), (3) Guinea savannah (a heavier type of woodland), (4) tropical rain forest or Guinea forest (thick climax vegetation) and (5) mangrove swamp (Akinyele 2009; Stenning 1957). Pastoralists practised dry season migration, moving to the southern (Guinea savannah) parts of the savannah zone to take advantage of better pasture there in the dry season.

Pastoralists’ cattle in the past could graze freely on vast and safe rangelands, reducing opportunities for their cattle to stray and graze on farmland. During the wet season, pastoralists returned to the northern savannah (Sudan savannah and semi-arid zone) to avoid the risk of tsetse - transmitted African animal trypanosomiasis. The symbiotic relationship between farmers and herders in the post-harvest period essentially consisted of cattle benefitting from crop residues in harvested fields and farmers deriving manure from dung deposited on their fields. There were trade opportunities as pastoralists purchased grain from crop farmers and crop farmers bought milk products from Fulani women. Even though pastoralists did not own land, they were welcome and encouraged to set up camp adjacent to farming communities who valued these opportunities (Stenning 1957; Blench 2003).

In the contemporary period, this symbiosis has been disrupted by increasing human and livestock populations, agricultural expansion, the introduction of inorganic fertiliser, the shift to fertiliser-based crops that produce no useful cattle residues (potato and maize), widespread availability of powdered milk and reduced demand for fresh milk. Farmers no longer require the traditional services of the Fulani pastoralists, but the Fulani pastoralists are still dependent on their goodwill for access to natural resources (Waters-Bayer and Bayer 1994; Blench 2004).

In the previous era of mutual dependency and abundance of resources, conflict between farmers and pastoralists occurred only during critical periods in the production calendar (sowing and harvesting). The relationship between different resource users was, for the most part, one of complementarity and peaceful co-existence. Scarcity of resources on which both farmers and herders depend has eroded this equilibrium and increased the likelihood for conflict of interest and competition. The effects of this scarcity have been distributed asymmetrically between farmers and pastoralists. Based on their status in the broader political context of Nigeria, some argue that pastoralists in the contemporary period have been denied access to resources and increasingly marginalised through landlessness and political powerlessness. Pastoralists therefore bear a larger burden of the scarcity than their rival group, the farmers, as a result of a gradual shift of power in favour of agriculturalists (Shettima and Tar 2008).

### Insecurity in and around Kaduna State

Independent Nigeria had enjoyed a period of relative peace until the two coups d’état in 1966 that ushered in military rule and civil war. The ensuing crises were a fusion of political, ethnic and religious disaffection and etched the pattern of future conflicts in Nigeria. The succession of military and civilian regimes that governed Nigeria until the late 1990s resulted in frequent coups and conflicts among ethnic and religious factions vying for supremacy. World Bank directives for the introduction of structural adjustment programmes in 1986 had severe and lasting negative effects on the economy (Babawale 2007). Many factories closed and industrial employment fell from 335,000 in 1985 to 27,000 in the 1990s with 49% of the population living below the poverty line (Babawale 2007). The incidence of long-lasting violent conflicts under military governance was low, due to the highly centralised governance and the swift crackdown on any such clashes by the army.

When Nigeria returned to democratic rule in 1999, several factors including decentralisation, the emphasis on indigeneity and local representation in governance and the rise of religious fundamentalism created the opportunities for violent tribal and religious conflicts. The northwest and north-central regions, with their high ethnic and religious diversity, have borne a large share of the violence (Nwozor 2014; UN 2009; NWGAV and AOAV 2015). The increase in rural inequalities between rich and poor/landless farmers and between rich ranchers and poor cattle owners, together with intense competition for scarce resources of land between these groups, has been a major cause of conflict in rural Nigeria. Outbreaks of violent conflict in and around Kaduna State have become commonplace since the return to democratic rule in Nigeria. Major conflicts include the following: the post-election violence of 2011; the Jos crises of 2001, 2004, 2008 and 2010; the Yelwa-Shendam crisis, 2004; the Kano reprisals for the Yelwa-Shendam crisis, 2004; the Kaduna Sharia and Miss World riots, 2000 to 2002; religious riots in Kano, 2004; and Sunni-Shia clashes in Sokoto, 2005, 2007 and 2010, and there have been long-running inter-communal clashes in Gwer-West, Benue State and Riyom/Barkin Ladi, Plateau State and southern Kaduna State (HRW 2005; NWGAV and AOAV 2015). Many of these conflicts were essentially spawned by competition for resources (e.g. land, cattle, education and lucrative political and professional positions) and exacerbated by a complex interplay of religion, ethnicity, politics and socio-economic disparities.

Rural conflicts have tended to focus on access to natural resource while urban conflicts have tended to be triggered by differences in ethnicity, religion, indigeneity and partisan politics. Disputes in urban areas have had direct impact on rural areas, the most important point of connection being replication in rural areas of the political, economic and religious sentiments and discourses that accumulate in cities during times of conflict (Higazi 2013; HRW 2001). In December 2010, for example, several churches were bombed in Jos during midnight mass and widespread riots followed with violence quickly spreading to rural areas on the Plateau. Most of this violence was directed against Muslims - Hausa and Kanuri residents in historic tin mining settlements and adjacent Fulani agro-pastoralist communities (Higazi 2011). Similarly, conflicts between herders and farmers in rural communities have escalated into widespread violence affecting urban areas (Roma 2008; Blench 2003; Higazi 2013; Moritz 2010). A minor incident at a rural abattoir (attack on a Christian butcher by a Muslim customer) resulted in rural-urban escalation of violence throughout Bauchi State, including the state capital in 1991 (Falola 1998).

Such conflicts have been compounded by the fact that Fulani have little or no land rights or rights of inheritance to pasture, water or cattle tracks in the country, meaning that the struggle for access to these resources underpins the increasingly strained relationships with the local indigene community.

### The development of grazing reserves

Grazing reserves emerged as a policy tool for modernisation of the livestock sector in Nigeria in the 1960s. The major aim was to provide pastoralists with more secure land tenure to encourage movement away from traditional transhumant cattle-rearing practices and reduce the clashes between pastoralists and crop farmers.

The Grazing Reserve Act of 1965 was passed by the Ahmadu Bello administration of the Northern Region (Waters-Bayer and Taylor-Powell 1986a; Awogbade 1987; Ingawa et al. 1989). In the third National Development Plan (1975 to 1980), the federal and state governments made a 120 million Naira (equivalent to USD$ 200 million at the time) investment in livestock development, of which 70% was allocated to grazing reserves. The management of this investment was overseen by the National Livestock Project Unit (NLPU), part of the Federal Livestock Department, which today is called the National Livestock Project Department (NLPD) (Ingawa et al. 1989). The NLPD was responsible for provision of general infrastructure including boreholes, dams, schools and roads. The formal gazetting of the KGR did not occur until 1996. The delay in gazetting the KGR together with lack of legalised land ownership and slow government investment in infrastructure dissuaded pastoralists from settling in the reserve (Waters-Bayer and Taylor-Powell 1986a). The failure of successive governments to promote the benefits of the grazing reserves and the persisting Fulani traditional dependency on transhumant practice also contributed to the initial reluctance of pastoralists to settle in the reserve.

The selection and acquisition of grazing lands is the responsibility of individual state governments, and the high levels of land compensation recommended by the Federal Land Use Act of 1978 (now Chapter L5, Laws of the Federation of Nigeria, 2004) dissuaded many states from setting up grazing reserves (Waters-Bayer and Taylor-Powell 1986a). At the start of the Second National Development Plan in 1970 to 1980, the grazing reserve concept became a national development strategy for cattle production and was reflected as the major pastoral development strategy in the Second, Third (1975 to 1980) and Fourth National Development Plans (1980 to 1985), as well as the First (1976 to 1983) and Second Livestock Development Programme FLDP (1976 to 1983) and SLDP (1987 to 1995). For example, the Third National Development Plan (1975 to 1980) proposed the establishment of a total of 22 million hectares in grazing reserves. By the end of 1977, (only) 2 million hectares had been acquired by both the state and federal governments (Gefu et al. 1988).

Officially, Nigeria now has 415 grazing reserves, but only one third are in use as the remainder have not been gazetted (IRIN 2009).

## Study area

The study was undertaken in the Kachia Grazing Reserve (KGR) in Kaduna State, northwest Nigeria, known as *Ladduga* (‘large bush’ or ‘wilderness’) by its Fulani inhabitants. The reserve lies between the urban centres of Kaduna and Zaria to the northwest, Jos to the southeast and Abuja to the southwest. The reserve, a total area of approximately 334 km^2^, lies within the sub-humid zone of Nigeria area and exhibits Northern Guinea-Savannah Woodland vegetation with an annual rainfall of 1000 to 1200 mm and an average temperature of 28 °C (Aregheore 2009). The wet season lasts between May/June and October while the dry season lasts from November to May/June.

The KGR was established to encourage sedenterisation of Fulani, aiming to improve livestock production and the standard of living of pastoralists, to promote access to social amenities and reduce farmer/pastoralist conflict (Waters-Bayer and Taylor-Powell 1986a, Gadoh 2011).

While the KGR reserve was ‘established’ by the Ministry of Animal and Forest Resources of the then North Central State (now Kaduna State) in 1968, developmental work did not begin until late 1970, when the Ministry of Agriculture mapped strategies for water and pasture development (Gadoh 2011). The KGR was re-officialised in 1988, after developmental work was carried out as part of a World Bank and State Government Intervention. The Kachia Grazing Reserve order was passed in 1996 and the land officially gazetted in 1996 (Kaduna State of Nigeria 1996).

At inception, the KGR was fumigated to eliminate the risks posed to cattle by tsetse-transmitted African animal trypanosomiasis (AAT) and was subsequently declared ‘tsetse free’ (Waters-Bayer and Taylor-Powell 1986a). The foundational infrastructure in the KGR included an administrative headquarters, roads, dams, bore wells, cattle dips, credit schemes, veterinary services and experimentation with various methods of pasture improvement as well as a smallholder dairy scheme supplying supplementary cattle feed on credit. The KGR served as a base for research into livestock production and disease control.

Any person wishing to settle in the KGR had to obtain a permit from the Project Officer which specified the number and type of animals permitted in the reserve, where they could graze, the size of the area allocated for grazing and a photograph of the permit holder (Kaduna State of Nigeria 1996). Each household settling in the KGR was allocated 10 ha: 4 for crop farming, 3 for fodder banks/feedlots and 3 for living quarters and livestock enclosures (Kaduna State of Nigeria 1996).

The KGR was initially well-staffed with 24 permanent employees including a range management officer and his assistant, four grazing control assistants, a veterinary assistant, a typist, a driver, three plant operators, five permanent and five casual labourers and two watchmen (Oxby 1984). Newer facilities include a secondary school, nine primary schools, a livestock-training centre, a community health clinic and a private health clinic.

The KGR is now mostly self-administered, with authority vested in the District Head and his council of elders (Okello et al. 2014). Many of the original facilities now have broken down, and one project officer remains on staff to handle administration, supervise the general activities of the reserve and provide monthly reports to the State Ministry of Agriculture. The Project Officer is also responsible for undertaking an annual census. Procedures to regulate and monitor the influx of people and their animals into the KGR have largely been abandoned due to a lack of human resources. Incoming families are reluctant to seek out the Project Officer as a fee is payable for a settlement permit that must be renewed annually. Instead, they seek approval from the District Head and farm as much land as they want, limited only by labour availability (Ducrotoy et al. 2017).

**Figure 1:**
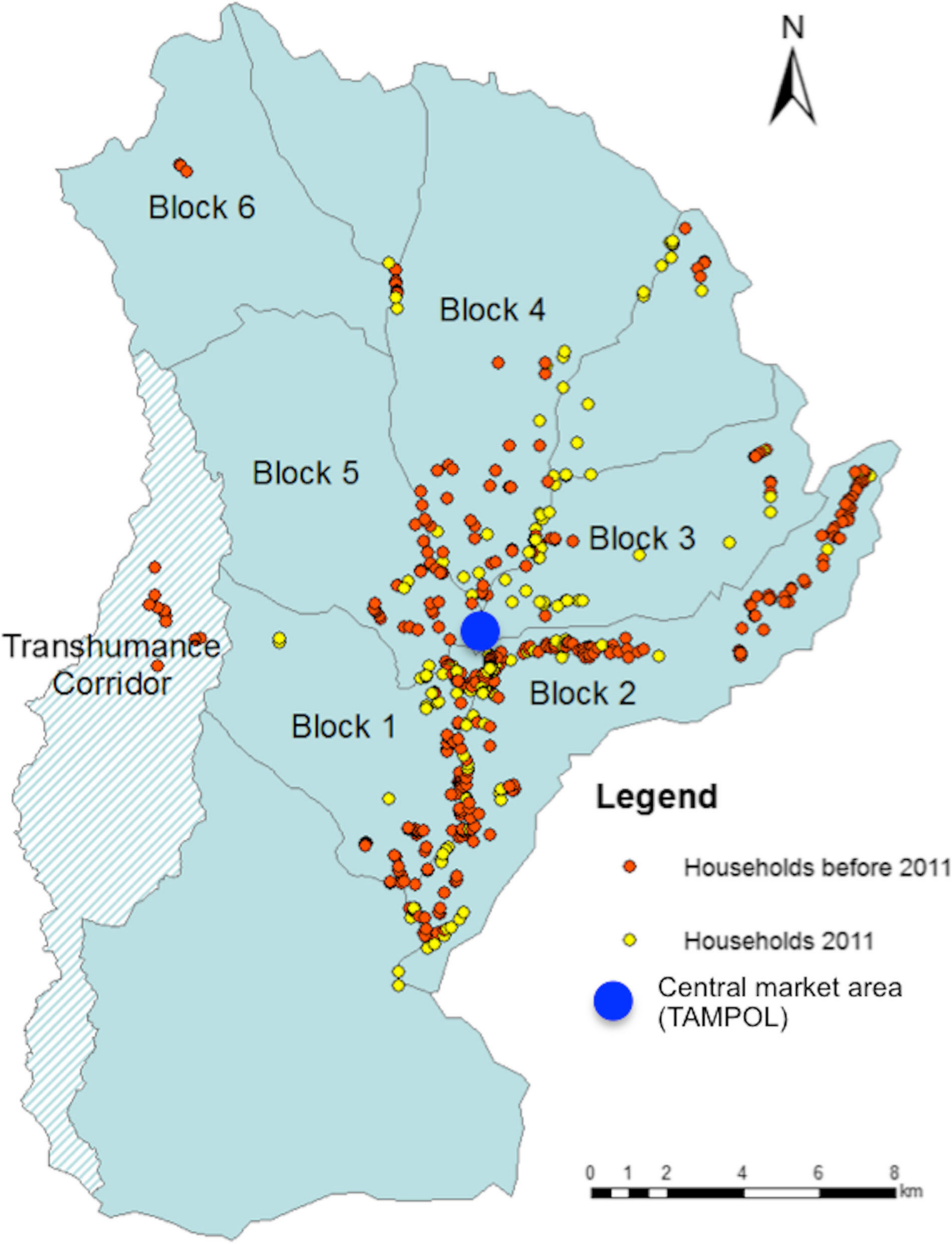
Geographical location of new immigrant (yellow) and established resident (red) households in June 2011

KGR settlers are exclusively Fulani pastoralists. The KGR is subdivided into six administrative blocks, which show slight variation in ecological and demographic characteristics (Ducrotoy et al. 2017) (Figure 1). The commercial hub of the KGR is referred to as *Tampol*, which was coined from the word ‘tarpaulin’. A tarpaulin was used to erect the initial temporary structures when the grazing reserve was first established. *Tampol* contains numerous shops (tea shops, butchers, etc.), and every Friday, as part of the special occasion of the *Salah al jama’ah* or Friday prayers, traders from the surrounding area come in and set additional stalls for business.

## Methods

This study complements a larger mixed methods study on zoonotic disease and pastoral livelihoods in KGR (Ducrotoy 2015, Ducrotoy et al. 2016 and Ducrotoy et al. 2017). Several rounds of data collection were undertaken as part of this study, including a complete household census in 2011, focus group discussions (FGDs) and key informant interviews (KIIs) undertaken from March to October 2011.

The census by the project team was facilitated by local participants: the Project Officer (state representative), who had 10 years of experience of working in the KGR, and a KGR community member to guide the census team through the difficult terrain, as the KGR is essentially a woodland area. Householders return to the KGR at the onset of the rains in June; hence, the census took place when all community members were present in the KGR, even if they practise dry season transhumance.

All 777 households in the reserve were geo-referenced by Global Positioning System (GPS) (Garmin Geko™), and data on household origin, year of settlement in the KGR, household and herd size and management were collected. Data on year of settlement in the KGR were used as a proxy for the number of households moving into the KGR over time. Motivation for settling in the KGR was verified directly with the respondents of new immigrant households during the census as well as during FGDs and KIIs.

The approach used to locate households was to firstly visit the *ardo* or village head of each village. The *ardo* then escorted the census team to every household in his village, promoting participation and ensuring a low non-response rate (only 25 households refused to answer questions, see Table 1). The *jewuro* or household head was the respondent of choice, followed by the next most senior male member of the household, as men have the most accurate knowledge of livestock numbers. Households were defined as *ruga*, consisting of a man, his wife or wives, unmarried children and dependents, as this was the unit of interest for the government. In this study, the term household corresponds to a *ruga*, as distinct from a *wuro*, which refers to the extended household or multiple *ruga* (Ducrotoy et al. 2017).

**Table 1:** New immigrants and previously established residents in 2011

	No. established residents	No. new immigrants	Total population	% increase
Households	503	249	752^a^	49.5
People	6142	2939	9081	47.9
Cattle	23,167	18,047	41,214	77.9
Sheep	5234	4921	10,155	94.0
Goats	3401	1427	4828	42.0

^a^Overall number of households was 777, but 25 households refused to provide information on year of settlement or number of people and livestock

Qualitative data were collected during FGDs and KIIs. The FGDs were conducted with *ardos* from all blocks to discuss immigration patterns into the KGR over time and explore differences between the blocks in terms of household and topographical characteristics. KIIs, broadly discussing past and future trends in the KGR, were undertaken with elderly, educated and elite male members of the community and young pastoralists. Topics explored during the FGD and KIIs included major events impacting on the KGR community in the last 40 years. Immigration into the KGR was discussed, including the motivations for pastoralists leaving their place of origin to settle in the KGR (push and pull factors of re-settlement). New immigrants, defined as households that moved to the KGR in 2011, were examined as a separate group to explore factors influencing immigration and differences to previously established residents of the reserve.

KGR residents are bilingual, speaking and understanding both Fulfulde and Hausa. Our interpreter spoke only Hausa, so focus group discussions were done in Hausa, the second language of KGR residents. Fulani terms for important words were recorded. Key informant interviews were undertaken in Hausa or directly in English when possible.

All census data were entered in Microsoft Excel®. Data were examined for inconsistencies or anomalies and cleaned to remove outliers and entry errors and checked for missing fields, and common answers entered with different spellings were checked. Completeness and precision checks were undertaken by cross-checking handwritten form data with Excel spread sheet data.

Qualitative data from FGDs and KIIs were analysed based on coding textual data into selected themes and sub-themes.

Data presented are census data; hence, significance tests or calculation of confidence intervals are redundant. Data were analysed using descriptive statistics and presented in tables, bar charts and line charts. The data is context- and time-specific, providing evidence of drivers and outcomes of Fulani immigration and integration before 2012.

## Results

### Demographics

The June 2011 census showed the KGR to be inhabited by 777 households. Individual household demographic data (available for 752 households) indicated a human, cattle, sheep and goat population of approximately 10,000, 40,000, 10,000 and 5,000 respectively. The average number of people, cattle, sheep and goat per household was 12, 55, 14 and 6 respectively (Table 1).

### New immigration in 2011

In total, 249 new households moved into the KGR in 2011. The number of households, people and goats in the KGR increased by almost 50%, while cattle numbers increased by 75% and sheep numbers almost doubled (Table 1). All new immigrants were Fulani pastoralists.

**Figure 2:**
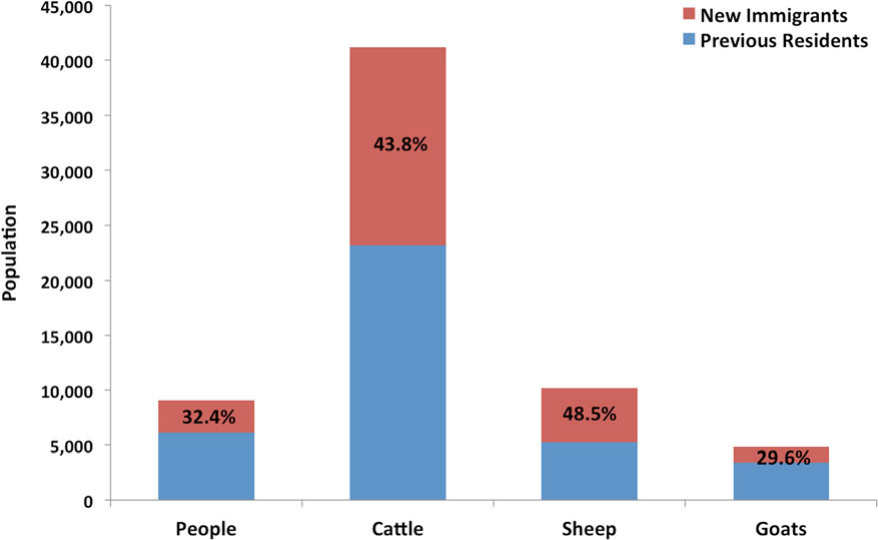
KGR populations in 2011 showing proportion of new immigrants

**Figure 3:**
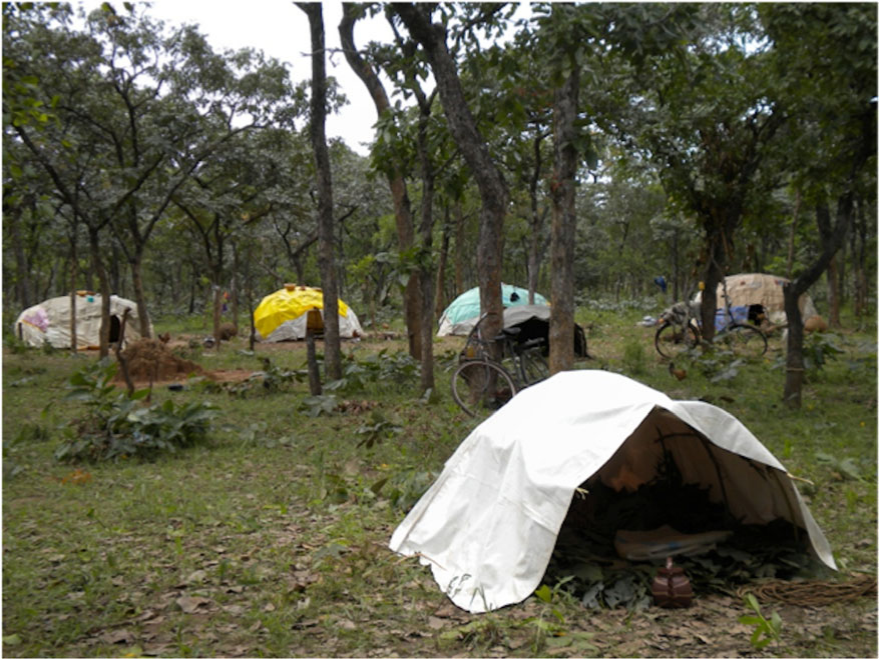
Temporary housing erected by families during the mass immigration event of April to May 2011

**Figure 4:**
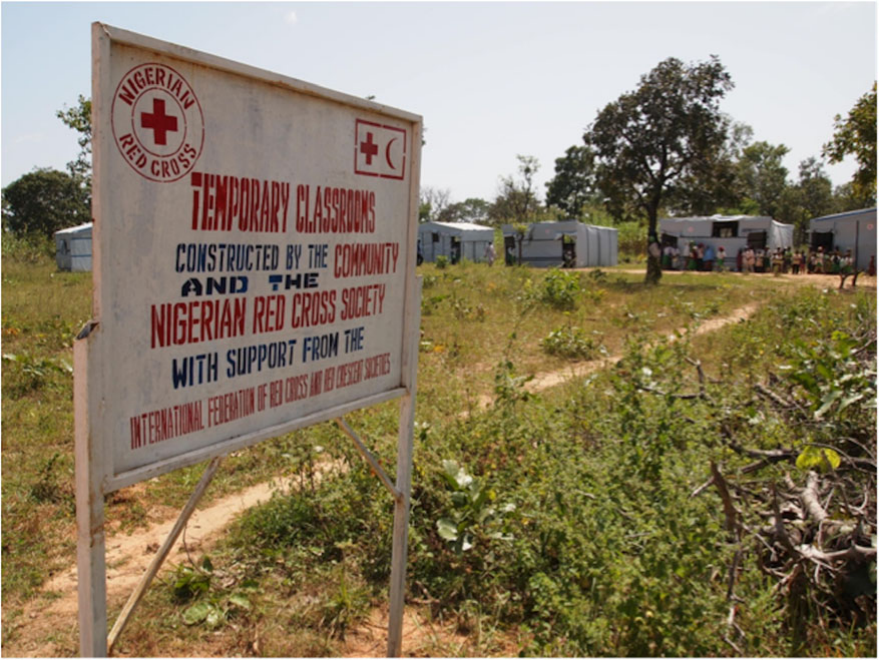
Temporary classrooms set up by the Nigerian Red Cross to cater for the refugee crisis in the KGR

The resulting 2011 population of humans and sheep within the KGR comprised approximately one third of new immigrants, who also accounted for almost half of the cattle and goat population as shown in Figure 2. Most of the new immigrants were internally displaced persons who arrived in April and May 2011 following post-election violence in Kaduna city and a spate of reprisal attacks targeting Fulani settlements across southern Kaduna State. Many immigrants had been burnt out of their homes and arrived with very few possessions other than their cattle and sheep. New immigrants settled throughout the blocks comprising the KGR (Figure 1) erecting temporary lodgings (Figure 3). Both the Red Cross and Nigeria’s National Emergency Management Agency (NEMA) provided relief materials and set up temporary infrastructure, for example, additional school classrooms (Figure 4).

### Immigration since 1978

**Figure 5:**
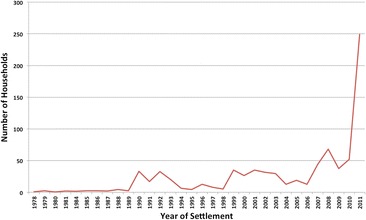
Number of households immigrating to the KGR 1978 to 2011

**Figure 6:**
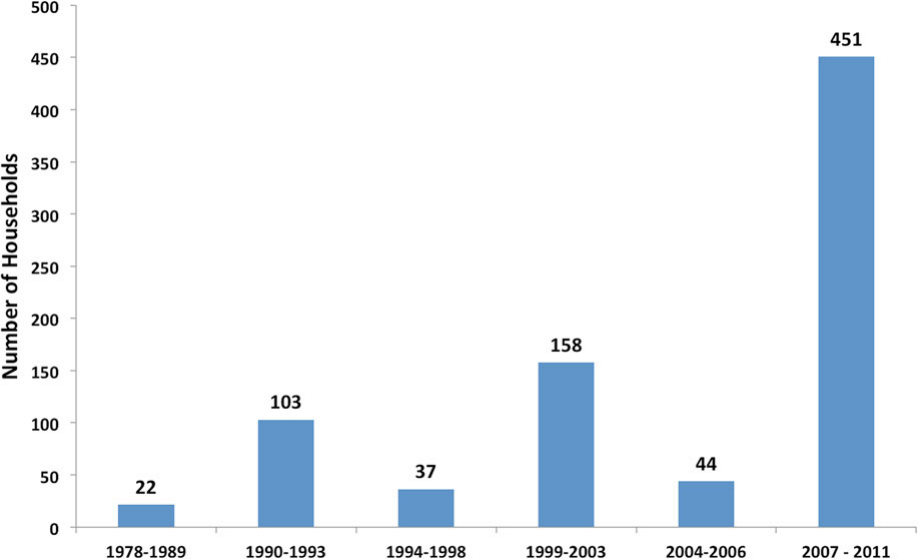
High and low periods of immigration

Mean immigration levels have risen steadily in the KGR from 6.4 households per year in the 1980s to 14.9 per year in the 1990s and 26.6 in the 2000s. In 2010, 52 households immigrated into the KGR followed by the 249 in 2011. In addition, there have been fluctuations in immigration levels, showing higher and lower periods of immigration (Figure 5). Peak immigration periods were 1990 to 1993, 1999 to 2003, 2008 to 2009 and 2010 to 2011 (Figure 6).

Periods of high immigration are associated with the incidence of violent conflicts in northwest and north-central Nigeria (Table 2). Push-pull factors responsible for the episodic rise in immigration into the KGR in the periods reflected in Figures 5 and 6 are discussed in the ‘Drivers of migration’ section.

**Table 2:** Proportion of immigrants from conflict areas during peak periods of immigration (IRBC 1993; Osaghae and Suberu 2005; Falola 1998; HRW 2003, 2001; IFRC 2011; Higazi 2013)

Period	Conflict	Location	Year	% immigrants from conflict area
1990 to 1993	Religious violence in Katsina by Shi’ite sect of Yahaya Yakubu	Katsina	1991	78
	Ethno-religious violence over abattoir use and revenue control in Bauchi	Bauchi	1991	
	Intra-Islamic religious violence stirred up by Izala sect in Kano	Kano	1991	
	Zangon-Kataf riots over market location and revenue control	Southern Kaduna	1992	
1999 to 2003	Ethno-religious clashes between Hausa-Fulani and southern Kaduna indigenes	Southern Kaduna	1999	64
	Protests against the introduction of Sharia	Kaduna	2000, 2001	
	Protests against ‘Miss World’ contest and related ‘blasphemous’ newspaper article	Kaduna, Abuja, Lagos	2002	
	Natural resource conflict, political and ethno-religious crisis between Hausa-Fulani ‘settlers’ and Plateau indigenes	Plateau	2001	
2007 to 2011	Natural resource conflict, political and ethno-religious crisis between Hausa-Fulani ‘settlers’ and Plateau Indigenes	Plateau	2008, 2010, 2011	78
	Election crisis of 2011 across Northern Nigeria	Kano, Kaduna, Bauchi, Gombe, Niger and Bornu	2011	
	At least 10 isolated ethno-religious clashes across six local governments	Southern Kaduna	2011	

**Figure 7:**
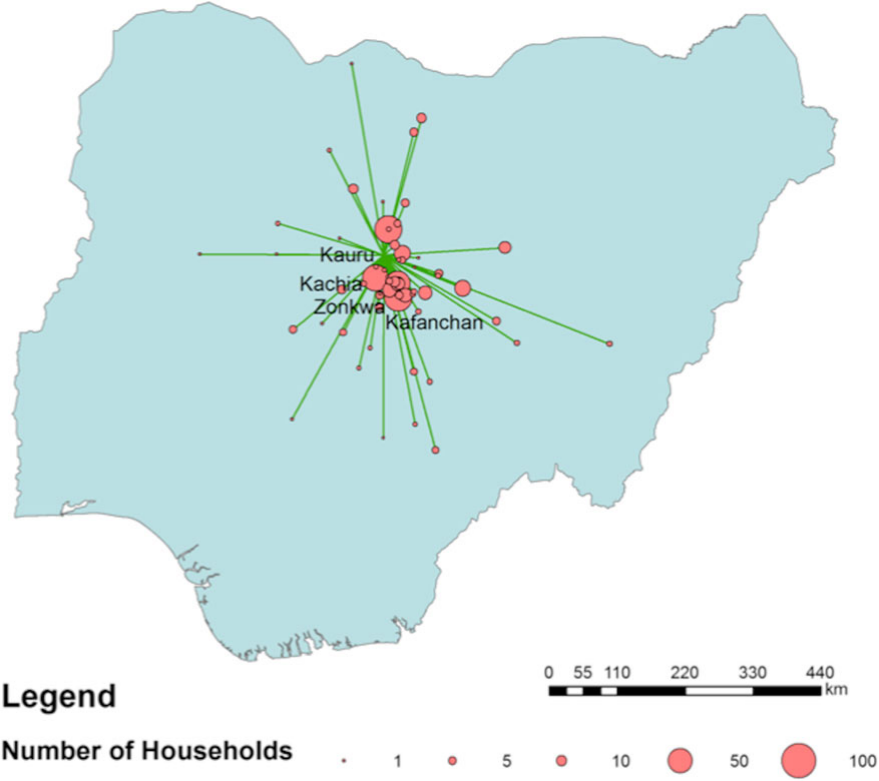
Map showing areas from which households have migrated

Most households that have settled in the KGR have come from a 400-km radius around the KGR, the neighbouring Bauchi State in the east, Plateau State in the southwest, Nassarawa State in the south and Niger State in the west. Others have migrated from Kano and Katsina States in the north (Figure 7).

### Family, flock and herd sizes per household

New immigrants had more cattle and sheep but fewer goats and slightly smaller families than established residents (Table 3).

**Table 3:** Mean human and animal population at level of household

Mean number per household	Established residents	New immigrants
Family size	12.3	11.9
Cattle	46.4	72.9
Sheep	10.5	19.9
Goats	6.8	5.8

### Emigration out of the KGR

Examination of grazing licences (available until the 1990s) and interviews with first settlers confirmed that most immigrant households relocated permanently to the KGR. An elderly *ardo* from Block 1, whose household moved into the KGR in 1978, stated that his block had only seen two households out of 131 (1.5%) move out of the KGR in his lifetime. FGDs corroborated the finding that households rarely left once they had settled in the KGR.

## Discussion

### Household and herd sizes

New immigrants possessed larger livestock holdings than the established agro-pastoralist KGR residents who had invested some of their assets in land and other cropping-related activities. This was supported from the FGD and KII, which indicated that many immigrant families brought with them cattle herds that were much larger than those generally kept by established residents. Established residents purported to keep smaller herds as a consequence of the limited carrying capacity and grazing resources in the KGR. Owners of larger herds routinely split these into multiple sub-herds, some of which are kept by relatives on holdings outside of the KGR (Ducrotoy et al. 2016; Ducrotoy et al. 2017). The ratio of sheep to goats maintained also supports this proposition - sheep are associated with nomadic life, while goats are more important to settled agro-pastoralists as they cannot be herded along with cattle.

This study supports the findings by Jabbar et al. (1995) who explored changes associated with sedentarisation and showed that recently settled pastoralists had considerably larger herds than long-term settlers. Herd size decreased gradually up to 10 years of settlement whereby it dropped sharply as households diversified their livelihoods by investing in cultivation, trade and paid employment (Jabbar et al. 1995; Azuwike and Enwerem 2010; Adunga 2013).

The observed differences in livestock numbers are also linked to access to natural resources. Sedentarisation is a trade-off between high-risk, high-opportunity livestock-reliant pastoralism and the lower risk, lower but more diverse opportunities of agro-pastoralism. More mobile pastoralists have greater access to natural resources and can relocate to where resources are most abundant, exploiting them more exhaustively. However, having no rights to land or natural resources, they are entirely dependent on the goodwill of their ‘host’ communities. Settled agro-pastoralists are limited by the resources available within their community which they must use responsibly and sustainably with their neighbours. They have the opportunity to acquire land and access rights to natural resources and to integrate with the host communities. Prevailing circumstances determine which way the balance shifts, with increasing pressure on natural resources currently favouring settlement and agro-pastoralism.

### Drivers of migration

The census data indicated four main periods of immigration into the KGR, the numbers increasing with time. During the first peak of immigration (1990 to 1993), 96 households entered the reserve. Between 1999 and 2003, 105 households moved to the KGR. In 2007 to 2010, 173 households moved into the reserve, and 2011 saw a large immigration of 249 households.

This study does not capture quantitative or qualitative data on households that might have left the reserve. The sample is therefore biased towards pastoralists who have stayed in the KGR. This bias is minimal, however, as emigration was confirmed to be a rare event.

Examination of grazing licences (available until the 1990s) revealed no record of anyone moving out, and key informant resident interviews with first settlers confirmed that most immigrant households relocated permanently to the KGR. This is common, since, once settled, pastoralists are reluctant to move (Gefu 1992). FGDs emphasised that once people move into the KGR, they very rarely move out, because, as one man stated: ‘we are at peace here’.

When asked why people moved to KGR, the unanimous answer was ‘to get away from conflict in other areas’. The KGR is seen as a safe haven, a legitimate area of land set aside for Fulani by the government, overcoming the land rights issues that confronted Fulani when living among hosting communities.

The conflicts the Fulani were seeking to avoid were of two types as reflected in the nature of the immigration. The first is a steady slow trickle of newcomers, gradually rising over the years. These are Fulani who are avoiding, mostly, non-violent natural resource conflicts and tensions arising from population increase and land pressure as well as antagonistic practices like the denial of herder access to land and water and the closure of cattle tracks and pastures by farmers. These restrictions convey the impression to the pastoralists that they are an unwanted nuisance (Majekodunmi et al. 2013). The second is sudden and massive immigration into the KGR by internally displaced people who fled from areas of violent conflicts and insecurity, often following personal experience of violence directed at and/or perpetrated by other Fulani.

These conflicts are rooted in competition and conflict over resources in both rural and urban areas but have strong political, ethnic and religious overtones. The peak immigration periods into the KGR coincided with notable periods of violence in northern Nigeria between 1990 and 2011.

The drivers for settlement in 1990 to 1993, 1999 to 2003 and 2007 to 2010 were confirmed during focus group discussions with community members, and the dates of peak immigration coincided with notable periods of rioting in northern Nigeria between 1990 and 2010 (Table 2). The first period of immigration in 1990 to 1993 was marked by the violence in Zangon-Kataf in 1992. Riots in Bauchi State were triggered by a minor incident at a small-town abattoir close to the state capital. The incident is estimated to have killed and injured numerous people and displaced 5,000 (Falola 1998). In May 1992, violence erupted in Zangon-Kataf as a result of a Muslim/Hausa versus Christian/Kataf conflict. Seventy-eight percent of the immigrants during this period came from the locations of these conflicts (Table 2), mostly from Kafanchan, Zangon-Kataf and Zonkwa in southern Kaduna (54%).

The early 2000s were marked by three major crises: the 2000 crisis of Kaduna, the Jos riots of 2001 and the 2002 Kaduna ‘Miss World Riots’. Fighting between Muslims and Christians in Kaduna in February to March 2000 followed a debate around the proposed introduction of Sharia Law in Kaduna State and resulted in the death of 2,000 people. The first Jos riot occurred in 2001 over the appointment of a Muslim politician and resulted in the death of over 1,000 people, destruction of infrastructure and homes and the displacement of between 50,000 and 200,000 civilians (HRW 2001). A further 250 people were killed in November 2002 when protests relating to the Miss World beauty contest due to be held in Nigeria degenerated into riots and widespread violence (HRW 2003). In 2008, a riot broke out in Jos over local elections and mounting tension between Muslim and Christian gangs left 300 persons dead and 10,000 displaced (Higazi 2013).

Sixty-four percent of households immigrating between 1999 and 2003 reported moving from Kaduna State (mostly Zangon-Kataf) and 19% from Plateau State (mostly Ganawuri). These numbers show that immigration into the KGR was predominantly due to the Jos and Kaduna crises, which started in urban centres and later spilled over into rural areas (Higazi 2013).

The majority of households (78%) that moved into the KGR between 2007 and 2010 came from conflict areas - 71% from surrounding southern Kaduna (mostly Zangon-Kataf (14%), Kauru (12%), Saminaka (8%), Kachia (8%), Kafanchan (7%)) and 7% from Plateau State. Violence and tensions in these areas was again confirmed to push people out of these areas during FGDs.

The post-election violence that erupted in April to May 2011 in the nearby areas of Zangon-Kataf, Kafanchana and Boto (southern Kaduna State) was the main driver for immigration into the KGR in 2011. The crisis in 2011 was marked by civil unrest and violent protests in the northern states of Kano, Kaduna, Bauchi, Gombe, Niger and Borno, before and after the presidential election in April 2011. The Nigerian Red Cross estimate that 75,000 people were displaced, 542 injured and 288 were killed during this period and that property worth hundreds of thousands of USD$ had been destroyed (IFRC 2011). Fulani herdsmen were singled out in the press as being responsible for some incidents - such accusations were not proven but fuelled unease and fear among the Fulani. Fulani settlements across southern Kaduna State were attacked and burnt, and survivors fled to the KGR (IFRC 2011). This is another example of urban violence in Kaduna city spreading to rural areas.

Northwest and north-central Nigeria is steeped in a long history of domination by the Muslim, Hausa-Fulani minority and suppression of the indigenous Christian majority during pre-colonial, colonial and post-independence regimes, which were all centralised governments. There have always been tensions in this area, with deep-seated resentment and contempt on both sides and sporadic outbreaks of violence over the years. The current dispensation of democratic, representative rule has seen the balance shift and majority groups assert themselves, leading to intense competition for authority and resources. It has been widely documented by reliable, impartial observers that both sides bear responsibility for inciting and perpetuating these clashes, along with the authorities (federal and state governments, police, military, traditional and religious leaders) whose interventions have often been slow, inappropriate and ineffectual (Nigeria Watch 2014; HRW 2005, 2011; Bocquier and Maupeu 2005; Kwaja 2011; IDMC 2012; Pew Forum on Religion & Public Life 2010; NWGAV and AOAV 2015; International Crisis Group 2012). Use of extreme force, extra-judicial killings and biases towards one side or the other have been the feature of police and military intervention in conflict situations (HRW 2002, 2010, 2013; Alemika 2013; Odinkalu 2008; NWGAV and AOAV 2015).

### Consequences of high immigration in the KGR

There is high population and grazing pressure in the KGR due to the increasing levels of immigration. Dry season cattle density was estimated at 10 head/km^2^ in KGR and 18 head/km^2^ and the wider sub-humid zone in the 1980s (Waters-Bayer and Taylor-Powell 1986a). The 2011 census shows a density of 123 head/km^2^ (41,214 cattle/334 km^2^). If one considers the original guidelines of allocating 10 ha per family (4 ha for crop farming, 3 for fodder banks/feedlots and 3 for living quarters and livestock enclosures) and the fact that only approximately 25% of the reserve is exploitable for growing crops and grazing cattle due to the rest being covered by thick forest (estimate from FGDs and KII with Project Officer), the 777 households present in 2011 were close to the maximum carrying capacity of 835 families.

The KGR is located on land with poor soil and pasture quality (Mohamed-Saleem 1986). The rise in cattle numbers from successive waves of immigrants arriving with larger and larger herds is not sustainable. The indigenous communities displaced by the creation of the reserve cooperated in releasing the land in the first place because it had the poorest soil (Oxby 1984). A scheme for pasture improvement and establishment of fodder banks initiated by the International Livestock Centre for Africa (ILCA) in the 1980s was not popular, never enforced and is now past recall by KGR inhabitants. Many households view extensive farming for home consumption and sale as the best use of land in the KGR. A mean of 5.2 ha is cultivated per household and one household farmed 50 ha (Ducrotoy et al. 2017). Inhabitants have significant investment in their cropping activities and make frequent use of hired labour and animal traction. Competition between cultivation and pasture within the reserve exacerbates pressure on grazing, replicating some of the natural resource conflict issues faced outside the reserve.

The fact that the KGR cannot support the cattle population within its boundaries forces pastoralists to undertake seasonal transhumance to sustain their cattle. In June 2011, 69% of households practised seasonal transhumance to source adequate pasture for their cattle. Of these, 31% practised dry season transhumance and 13% wet season transhumance and 26% practised both wet and dry season transhumance. By October 2011, 90% of households reported having taken their cattle on dry and/or wet season transhumance during the previous 12 months from grazing pressures caused by the influx of refugees in June 2011 (Ducrotoy et al. 2016).

Dry season transhumance is a long-established practice of pastoral life. However, wet season transhumance is a more recent development both in the KGR and in Nigeria more generally and a direct response to reduction in grazing caused by land pressure. A recent study in 30 villages in Jos revealed that 73% of herders practised transhumance with over half practicing both wet and dry season migration. The major driver of wet season migration was limited availability of resources and attendant restricted access to them (Majekodunmi et al. 2014).

Transhumance data from pastoralists living in the KGR (Ducrotoy et al. 2016) and Jos Plateau (Majekodunmi et al. 2014) offer an insight into the nuances of land pressure and pasture quality as drivers for migration, especially during the wet season, in and outside of a grazing reserve. Most households in the KGR interviewed in June 2011 (83%) described poor quality of grass, scarcity of water and animals running out of pasture (overgrazing) as the main push factors for transhumance. A minority (12%) linked migrating to tsetse fly challenge. Only 5% reported undertaking transhumance because ‘it is a custom or tradition’ or ‘attachment to the thrill of grazing in a new environment’.

In Jos, the inability of Fulani to control natural resources and poor access to these, especially during the wet season, highlighted social factors as drivers of migration. Lack of water and grass was a motivator for transhumance in only 54% of villages. Lack of access to resources was sub-categorised into lack of access to land (35%), bush burning (9%) and conflict with farmers (2%).

Pastoralists in the KGR have access to pasture and water, but soil and pasture quality is poor, driving dry season transhumance: ‘Usually everyone who went on dry season migration brings their cattle back to the KGR as soon as they have word that the rains have come to KGR’ (FGD discussant). The arrival of the new immigrants caused a shift in this paradigm. Mass influx of people and cattle on a restricted and already saturated land resulted in animals running short of pasture (overgrazing). ‘In previous times, when there were less people in KGR, the grass had time to recover during the wet season, but now the good grass is being replaced by dust or weeds which the cattle cannot eat’ (KII, village head).

Social factors were also at play as new immigrants were used to better conditions and had larger herds and were therefore willing to move even during the wet season. This statement from a KGR focus group discussant illustrated the main reason given for wet season transhumance: ‘when newcomers came they discovered that KGR has limited grasses, and they were more motivated to go on transhumance because they have come from areas which had a lot of grass’.

In the KGR, transhumance practice was mostly determined by herd size. Herds with less than 50 animals could be sustained within the KGR, but a sub-group of cattle from larger herds had to leave the KGR to find supplementary grazing. Duration of settlement influenced when and how far households moved their cattle. Dry season transhumance practice was equally undertaken among new and old inhabitants, but wet season transhumance was practised mostly by recent settlers and new immigrants, who also travelled farther from the KGR when on transhumance (Ducrotoy et al. 2016). ‘Most of those that go far away are new settlers or newcomers that have come in the last two years, those of us that have settled here for a long time do not go far’.

New arrivals had recently come from areas of more abundant, higher quality pasture and were more motivated to travel in search of better grazing. Long-term residents are used to the conditions in and around the KGR and are content with available resources. FGDs also revealed the community welcomed the new immigrants ‘with open arms’, as ‘they are our kin’ (FGD participant), but during the course of the study, there were signs of mounting tension over competition for the shrinking pasture. Poorly managed and too few grazing reserves as well as shrinking resources tend to fuel competition among KGR pastoralists.

The fact that a significant proportion of KGR residents had been personally affected by violence and lost property and family profoundly affected their perception of new immigrants. Researchers recall a woman exhibiting profound distress when asked if her husband, the *jewuro*, could give information about her household; he had recently been killed in the post-election clashes of April 2011. The world outside the reserve is generally viewed with deep suspicion and a strong sense of persecution. The zoonotic disease survey was almost derailed because the field manager was from Plateau State and several KGR residents had fled the Plateau during the 2001 crisis (Ducrotoy et al. 2015). All work was put on hold for three months and it took the intervention of a senior civil servant, a Fulani, to get community leaders’ approval for work to continue. This shows that although grazing reserves improve the lot of pastoral people and solve many natural resource use issues, they can foster stereotypes, suspicion and ignorance of ‘outsiders’ (Ducrotoy et al. 2015).

Grazing reserves are struggling to meet the needs of pastoralists in Nigeria today (Okeke 2014). The incidence of violent farmer-herder clashes has reached intolerable levels and is an issue of national concern. In 2012, the Fulani appealed to the federal government and a grazing reserve (National Assembly 2011) was proposed to appropriate land for grazing in all 36 states of the federation. This was met by furious opposition across Nigeria, but with particularly strong opposition from the Middle belt. The main arguments were that other Nigerian citizens can only acquire land by inheritance or purchase and pay for premises and inputs to run their businesses, and there was reluctance for Fulani to be given these assets for free. The question was raised ‘why should government appropriate land from other Nigerians to support Fulani livelihoods?’, and the feeling was that they should lease or purchase land for pasture and that the government could support them by overseeing such negotiations (Okeke 2014). This bill and two others (one duplicating and the other in direct opposition to it) were withdrawn by the senate in 2016 as they were judged unconstitutional. Instead, the House of Assembly committee recommended reclamation and development of all current grazing reserves under the 1965 Grazing Reserve Act (albeit that most were recognised as being severely encroached), clear demarcation and enforcement of cattle routes and support for cattle production within these protected areas. These recommendations are being implemented by some state governments with successful reduction of clashes (Okello et al. 2014).

## Conclusions

This study has shown how violent and non-violent conflict is the main driver for immigration into the KGR. Increasing insecurity in northwest Nigeria and corresponding high immigration levels have led to population pressures within the reserve.

Since the governance of the KGR is now mostly by customary authorities (Okello et al. 2014), the residents of the grazing reserve have shifted land use from pure grazing to a mixed-use area comprising grazing, cultivation, internally displaced persons camp and resettlement zone.

Settlement in grazing reserves does not prevent transhumance practice. Pastoralists settle and become agro-pastoralists, but transhumance persists and may even increase. From an ecological point of view, transhumance is a highly effective and appropriate way to use grazing resources that vary in space and time. In Nigeria today, given the insecurity and politicisation of herder-farmer conflicts, it can also have economic and social disadvantages. Whole families no longer move together as a unit, as only young men go on transhumance, impacting on family and social life. Tension and insecurity means transhumant herders and cattle are at risk from cattle rustlers and from clashes with farmers. Herd splitting and long periods of transhumance mean less milk for home consumption and reduced income for pastoralist women. The costs sometimes overtake the benefits, for example, 54% of production costs for Jos Fulani went into paying experienced hired herders to protect cattle on transhumance (Majekodunmi et al. 2014).

Mobility has increased as more households are forced into transhumance for longer periods. It is the interplay between availability and access to pastoral resources which drives transhumance. Wet season transhumance in the KGR peaked with the arrival of new immigrants due to ensuing competition for grazeable land.

Although steps are being taken to address these conflicts, security will not be restored overnight and immigration to the KGR will continue to rise. Strategic sustainable intervention is required both within and outside the KGR to ease land pressure and secure rural livelihoods. Pastoralists themselves are already working on solving these problems, by going on transhumance and distributing the grazing pressures over the available resources. But this coping mechanism is unsustainable and also part of what causes the insecurity, by increasing opportunities for farmer-herder clashes.

The pervasive natural resource conflict issues must be addressed. The House of Assembly committee recommendations with regard to the reclamation and development of grazing reserves under the 1965 Grazing Reserve Act and clear demarcation and enforcement of cattle routes represent efforts to balance the inequality in land tenure administration and will be important in reducing conflict. Pastoral self-governance networks are known to successfully regulate grazing habits (Okello et al. 2014). These networks will be very helpful in promoting pasture development.

Investment in and enforcement of pasture development is key, and activities such as water and soil conservation and improvement and planting fodder/browse species must be prioritised by both government and pastoral authorities. In the complex landscape of farmer-herder conflicts, transhumance is one of the root causes of insecurity. Pasture development and provision of transhumance corridors could impact on insecurity by reducing or re-channelling transhumance to demarcated zones.

## Abbreviations

FGD: Focus group discussion
IRIN: Integrated Regional Information Network
KGR: Kachia Grazing Reserve
KII: Key informant interview
NLPD: National Livestock Project Department
NLPU: National Livestock Project Unit
USD$: US dollar

## Authors’ information

MJD is a veterinarian and transdisciplinary postdoctoral researcher at the University of Edinburgh (UoE) working on zoonotic disease control in Africa at the time of study and now project managing animal health and production projects in Africa and Asia at Ceva Sante Animale. AM is an epidemiologist and postdoctoral researcher at the UoE at the time of the study now working at the University of Ghana. APMS is a livestock and health economist. WJB, AMG and RAO are veterinarians and brucellosis researchers at National Veterinary Research Institute, Vom, Nigeria. HB is a junior livestock researcher at UoG. SW is group leader of the Neglected Zoonoses Research Group at UoE.
